# Feedback control of the CXCR7/CXCL11 chemokine axis by estrogen receptor α in ovarian cancer

**DOI:** 10.1002/1878-0261.12362

**Published:** 2018-08-23

**Authors:** Samira Benhadjeba, Lydia Edjekouane, Karine Sauvé, Euridice Carmona, André Tremblay

**Affiliations:** ^1^ Research Center CHU Sainte‐Justine Montréal Canada; ^2^ Department of Biochemistry and Molecular Medicine Faculty of Medicine University of Montreal Canada; ^3^ CHUM Research Center Institut du Cancer de Montréal Canada; ^4^ Centre de Recherche en Reproduction et Fertilité University of Montreal Saint Hyacinthe Canada; ^5^ Department of Obstetrics & Gynecology Faculty of Medicine University of Montreal Canada

**Keywords:** ACKR3, chemokine receptors, ESR1, estrogen receptors, ovarian cancer mesenchymal subtype, stromal compartment

## Abstract

Ovarian cancer (OC) is one of the most intractable diseases, exhibiting tremendous molecular heterogeneity and lacking reliable methods for screening, resulting in late diagnosis and widespread peritoneal dissemination. Menopausal estrogen replacement therapy is a well‐recognized risk factor for OC, but little is known about how estrogen might contribute to this disease at the cellular level. This study identifies chemokine receptor CXCR7/ACKR3 as an estrogen‐responsive gene, whose expression is markedly enhanced by estrogen through direct recruitment of ERα and transcriptional active histone modifications in OC cells. The gene encoding CXCR7 chemokine ligand I‐TAC/CXCL11 was also upregulated by estrogen, resulting in Ser‐118 phosphorylation, activation, and recruitment of estrogen receptor ERα at the CXCR7 promoter locus for positive feedback regulation. Both CXCR7 and CXCL11, but not CXCR3 (also recognized to interact with CXCL11), were found to be significantly increased in stromal sections of microdissected tumors and positively correlated in mesenchymal subtype of OC. Estrogenic induction of mesenchymal markers SNAI1, SNAI2, and CDH2 expression, with a consequent increase in cancer cell migration, was shown to depend on CXCR7, indicating a key role for CXCR7 in mediating estrogen upregulation of mesenchymal markers to induce invasion of OC cells. These findings identify a feed‐forward mechanism that sustains activation of the CXCR7/CXCL11 axis under ERα control to induce the epithelial–mesenchymal transition pathway and metastatic behavior of OC cells. Such interplay underlies the complex gene profile heterogeneity of OC that promotes changes in tumor microenvironment and metastatic acquisition.

**Abbreviations**
DMEM: Dulbecco's modified Eagle's medium
DPN: diarylpropionitrile
ECM: extracellular matrix
EMT: epithelial–mesenchymal transition
ERE: estrogen‐responsive elements
GSEA: gene set enrichment analysis
OC: ovarian cancer
PGR: progesterone receptor
PPT: propylpyrazole triol
SNAI1: snail family zinc finger 1
SNAI2: snail family zinc finger 2
TCGA: The Cancer Genome Atlas

## 1 Introduction

Ovarian cancer (OC) is the most aggressive and deadliest of gynecologic malignancies, mainly because of its high metastatic potential and late diagnostic due to lack of early tumor biomarkers. Preclinical studies have shown that factors involved in development and progression of OC include sex steroid hormones. Indeed, hormone replacement therapy based on exogenous estrogen given to postmenopausal women increases their risk to develop OC (Beral *et al*., 2007; La Vecchia, 2017; Malvezzi *et al*., 2016; Zhou *et al*., 2008). Estrogens are well known to be oncogenic in breast cancer by regulating numerous genes involved in development, proliferation, and progression of the disease. Gene regulation by estrogen is mediated by the estrogen receptors ERα (NR3A1) and ERβ (NR3A2), members of the nuclear receptor family of transcription factors (Mangelsdorf *et al*., 1995). Upon estrogen binding, the ERs initiate transcription by directly binding to estrogen‐responsive elements (ERE) contained in target genes promoters. ERα is expressed in up to 60% of ovarian epithelial tumors with higher levels compared to normal ovaries although its prognostic value remains uncertain (Chan *et al*., 2008; Hildebrand *et al*., 2010; Sieh *et al*., 2013). Despite also a high expression of ERβ in OC, its significance in terms of tumor subtypes and isoform specificity is variable (Ciucci *et al*., 2014; Kyriakidis and Papaioannidou, 2016).

The progression of OC toward angiogenic and metastatic stages has been associated in part with chemokine signaling pathways. Stromal‐derived factor SDF‐1 (CXCL12) and its G protein‐coupled receptor CXCR4 are highly expressed in OC and associated with poor outcome (Guo *et al*., 2014; Liu *et al*., 2014; Popple *et al*., 2012). The recent identification of CXCR7 (ACKR3) as a second receptor for SDF‐1 has made essential to re‐evaluate the response to SDF‐1 in pathophysiology (Balabanian *et al*., 2005). In addition, CXCR7 is also activated by interferon‐inducible T‐cell alpha chemoattractant I‐TAC (CXCL11), adding to the complexity of CXCR7 cellular responses (Burns *et al*., 2006). CXCR7 is expressed in the hematopoietic system, heart, vascular endothelial cells, bone, kidney, and brain and plays a pivotal role in cell growth, survival, and migration (Burns *et al*., 2006; Sanchez‐Martin *et al*., 2013; Thelen and Thelen, 2008). Several studies have implicated a tumorigenic role of CXCR7 in various cancers, including breast, lung, and prostate, with increased growth, migration potential, and prognostic significance (Miao *et al*., 2007; Saha *et al*., 2017; Wu *et al*., 2015). Conversely, CXCR7 expression does not always correlate with poor outcomes, such as in neuroblastoma and colon cancer (Heckmann *et al*., 2014; Liberman *et al*., 2012), suggesting context‐dependent actions of CXCR7. However, the role of CXCR7 in OC is less characterized. CXCR7 expression was identified in neoplastic ovaries (Jaszczynska‐Nowinka *et al*., 2014), and ligand activation of CXCR7 was shown to induce matrix metalloproteinase MMP‐9 in epithelial OC cells (Yu *et al*., 2014). In addition, CXCL11 and CXCL12 expression levels were found augmented in ovarian carcinomas compared to normal ovaries (Furuya *et al*., 2007; Jaszczynska‐Nowinka *et al*., 2014). However, the signaling events regulating gene expression of CXCR7 and its ligands in OC cells remain unknown.

Previous findings have identified estrogen as a positive regulator of the CXCL12/SDF‐1 gene in breast cancer cells (Hall and Korach, 2003; Sauve *et al*., 2009), and we have described that such regulation is part of a positive autocrine feedback loop involving CXCR4 and estrogen receptors to promote cell growth (Sauve *et al*., 2009). In this study, we identified CXCR7 as an estrogen‐responsive gene whose expression is specifically enhanced in ER‐positive OC cells through direct recruitment of ERα and favorable chromatin modifications at the CXCR7 promoter. We also show that I‐TAC/CXCL11 expression is enhanced by estrogen, resulting in phosphorylation of ERα and feedback regulation of the CXCR7 gene. Our findings identify a regulatory loop between ERα and CXCR7 chemokine axis, promoting optimal response to estrogen and invasion phenotype to OC cells.

## 2 Materials and methods

### 2.1 Cell culture and treatments

Human OC OVCAR‐3 and SKOV‐3 cells, human breast cancer MCF‐7 and MDA‐MB‐231 cells, and human endometrial cancer HEC‐1A and Ishikawa were maintained in Dulbecco's modified Eagle's medium (DMEM; Sigma, Oakville, ON, Canada) supplemented with 10% FBS (Wisent Inc., St‐Bruno, QC, Canada). Human epithelial OC TOV21G cells were derived from clear cell carcinoma and cultured in OSE medium supplemented with 10% FBS (Yoffou *et al*., 2011). TOV2295 and TOV3133G cell lines were derived from high‐grade serous carcinomas and cultured as described (Letourneau *et al*., 2012). Prior to treatments, cells were seeded in phenol red‐free DMEM supplemented with dextran‐coated charcoal‐treated FBS. Cells were treated with 17β‐estradiol (E2) and ICI 182,780 obtained from Sigma, I‐TAC and SDF‐1 from Cell Sciences Inc., (Newburyport, MA, USA) and specific agonist propylpyrazole triol (PPT) for ERα and diarylpropionitrile (DPN) for ERβ obtained from Tocris.

### 2.2 RNA isolation and quantitative PCR

Total RNA was extracted from cells, and complementary DNA was prepared and subjected to real‐time PCR as described (Rodrigue‐Way *et al*., 2014). Values are derived from at least three separate experiments performed in triplicate and normalized to ribosomal protein RPLP0 or to β‐actin expression.

### 2.3 Western blot analysis

Cells were harvested, and immunoblotting was performed as described (Sanchez *et al*., 2013). Antibodies for ERα, ERα‐pSer118, pErk1/2, and Erk1/2 were from Cell Signaling, and ERβ and epithelial–mesenchymal transition (EMT) markers from Santa Cruz Biotech (Mississauga, ON, Canada). In each experiment, total protein loading was normalized using an anti‐β‐actin antibody (Novus Biologicals, Littleton, CO, USA).

### 2.4 GEO and TCGA gene expression data

Gene expression data (GSE40595, GSE38666, and GSE9890 profiles) were retrieved as raw signals from GEO datasets (http://www.ncbi.nlm.nih.gov/gds), analyzed, and log2 scaled using the GEO2R online analysis tool (http://www.ncbi.nlm.nih.gov/geo/geo2r). Correlation and distribution analysis was performed in human OC samples using data from the Cancer Genome Atlas (TCGA) cohort. Expression data were retrieved from the cBioportal for Cancer Genomics (http://www.cbioportal.org) using the high‐grade serous OC dataset (Cancer Genome Atlas Research Network, 2011). Stratification into the various OC subtypes was based on specific gene signature of the TCGA dataset (Cancer Genome Atlas Research Network, 2011; Konecny *et al*., 2014). Gene expression was normalized to the distribution of each gene in tumors and annotated as *z*‐scores as described (Edjekouane *et al*., 2016). The mesenchymal signature score was derived from a subset of overlapping genes from the TCGA mesenchymal OC subtype and the Tothill C1 (tumor desmoplasia) subtype samples that were positively correlated with mesenchymal markers and extracellular matrix (ECM) remodeling (Verhaak *et al*., 2013; Yang *et al*., 2017). graphpad prism 6 (La Jolla, CA, USA) was used to perform Pearson correlation test (two‐tailed), and significance was set at *P* < 0.05.

### 2.5 ChIP assay

ChIP assays were performed as described (Edjekouane *et al*., 2016; Sanchez *et al*., 2013; Sauve *et al*., 2009). Cells were treated with vehicle (ethanol), 10 nm estradiol, or 50 nm I‐TAC for 45 min, and chromatin was harvested and analyzed by ChIP‐qPCR using antibodies as described (Edjekouane *et al*., 2016). Preimmune IgG was used as a negative control. Each analysis was performed in duplicates, and results are derived from at least three independent ChIP experiments.

### 2.6 ChIP‐seq analysis

The ChIP‐seq data were generated and processed as previously described (Edjekouane *et al*., 2016) with the sequenced reads aligned against the human reference genome hg38 and visualized with IGV. ERα ChIP‐seq data from Ishikawa cells were obtained from the ENCODE consortium (ENCSR000BIY).

### 2.7 CXCR7 promoter constructs and mutagenesis

The CXCR7 proximal promoter regions were amplified by PCR according to GenBank sequence of the CXCR7/ACKR3 gene contig (NT_005120) and the UCSC hg38 genome assembly. Amplified fragments corresponding to proximal P1 (2610 bp) upstream from the transcriptional start site and P2 promoter (2532 bp) located in intron 1 were inserted in front of the luciferase coding region in pBLuc plasmid as described (Sanchez *et al*., 2013). P2 truncated fragments were generated by restriction digest with Pst1 (P2Δ1) and HindIII (P2Δ2). Site‐directed mutagenesis of the ERE‐42463 was performed by PCR. All constructs were validated using automated sequencing.

### 2.8 Transfection and luciferase reporter assay

Transfection of cells and luciferase assays were performed as described (Picard *et al*., 2012). Treatments were usually for 16 h otherwise stated. Cells were then harvested in potassium phosphate buffer containing 1% Triton X‐100 and lysates analyzed for luciferase activity using a plate reader (Perkin‐Elmer). Luciferase values were normalized for transfection efficiency to β‐galactosidase activity and expressed as relative fold response compared with controls. Data were derived from at least three independent experiments performed in triplicates.

### 2.9 RNA interference

CXCR7 knockdown was performed by infecting OVCAR‐3 cells with shRNA‐carrying lentiviral particles as described (Rodrigue‐Way *et al*., 2014). Flow cytometry sorting was performed to select cells stably transduced with lentivirus targeting CXCR7 (sh‐CXCR7) and compared to shRNA‐negative control (shCtl). Two independent shCXCR7 cell lines were generated using CXCR7 targeted sequences GCATCTCTTCGACTACTCAGA and CGCTCTCCTTCATTTACATTT. Efficient knockdown was monitored by quantitative RT‐PCR and western blot analysis.

### 2.10 Cell migration assay

OVCAR‐3 cells were seeded (10^5^ cells/well), and when 80–90% confluence was reached, linear wounds were made using a sterile 10 μL pipette tip. Cells were rinsed with PBS and treated with 10 nm E2 or vehicle (EtOH) freshly added every 24 h in culture medium. Wound images were then recorded over a period of 48 h, and wound closure area was measured using imagej software (https://imagej.nih.gov/ij). Data were calculated as % wound healed vs. 0 h derived from duplicate wells for each condition obtained from three independent experiments.

### 2.11 Statistical analyses

Values are expressed as the means ± SEM (standard error of the mean), derived from at least three independent experiments performed in triplicates. Single comparisons between two groups were determined by Student**’**s *t*‐test. Comparisons between multiple groups were determined by one‐way ANOVA followed by Bonferroni post‐test. *P* values < 0.05 were considered significant.

## 3 Results

### 3.1 CXCR7 is strongly expressed in human ovarian cancer cells and tumor stroma

**Figure 1:**
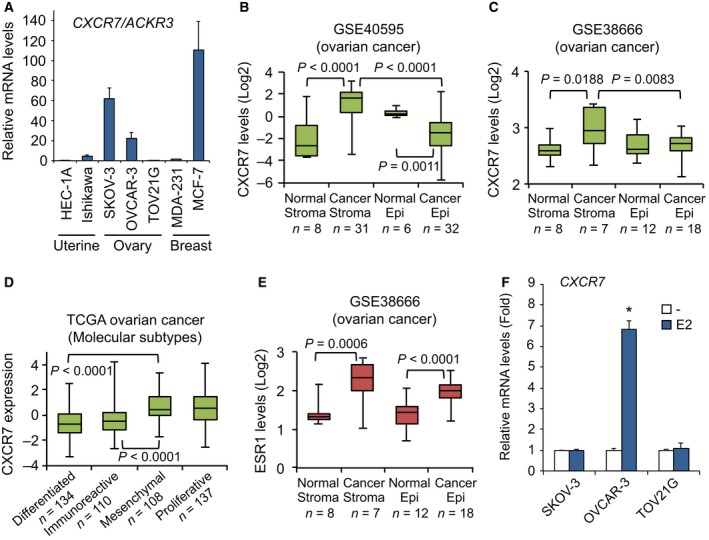
CXCR7 is strongly expressed in ERα‐positive gynecologic cancer cells and tumor stroma. (A) A panel of uterine, ovary, and breast cancer cell lines was analyzed for CXCR7 mRNA expression by qPCR. Each expression value was quantified to RPLP0 expression. Data are representative of at least three independent experiments performed in triplicates. Error bars represent SEM. (B) Boxplots showing the expression levels of CXCR7/ACKR3 in microdissected normal and tumor epithelium with respective matched surrounding stromal sections included in the ovarian dataset GSE40595. (C) Boxplots of the expression level of CXCR7 in microdissected normal and tumor samples of GSE38666. (D) Boxplots showing the relative CXCR7 expression in the various molecular subtypes of the ovarian TCGA dataset. (E) Boxplots of the expression level of ESR1 in microdissected normal and tumor samples of GSE38666. (F) Estrogen‐regulation of CXCR7 gene expression. Cells were treated with 10 nm 17β‐estradiol (E2) or ethanol vehicle (–) for 16 h, and then harvested for CXCR7 expression as in A. Data were analyzed using Student's *t*‐test and are representative of at least three independent experiments performed in triplicates. Error bars represent SEM. *P < 0.001 versus respective vehicle‐treated cells.

To investigate the expression pattern of CXCR7 in reproductive cancer cells, we tested a subset of cancer cell lines derived from human uterine, ovary, and breast tumors. High levels of CXCR7 mRNA were found in ovarian SKOV‐3 and OVCAR‐3 cancer cells, and in breast MCF‐7 cancer cells, and to a lesser extent in uterine Ishikawa cells, compared to the other cell lines which exhibit very low expression levels (Fig. 1A). The CXCR7 expression pattern strikingly correlates with the ERα status of cells with elevated ERα protein levels found in OVCAR‐3, MCF‐7, and Ishikawa cells, in contrast to HEC‐1A, TOV21G, and MDA‐MB‐231 cells (Fig. S1A). SKOV‐3 cells exhibit low levels of ERα, but these cells have been described as not having a functional ERα (Lau *et al*., 1999), which might suggest that high levels of CXCR7 might be independent of ERα. To further define the expression pattern of CXCR7 in epithelial OC, we next analyzed a microdissected profile of human isolated surface epithelium ovarian tumors and a matched set of surrounding cancer stroma. We found a significantly higher expression of CXCR7 in tumor stroma sections when compared to normal stroma or to tumor epithelium compartments (Fig. 1B). Further validation of increased stromal CXCR7 was also provided in two other independent profiling datasets of OC (Fig. 1C and Fig. S1B). In addition, analysis of stratified subtypes of OC based on specific gene signature of the TCGA dataset (Cancer Genome Atlas Research Network, 2011; Konecny *et al*., 2014) indicates a significant increase in CXCR7 gene expression in the mesenchymal and proliferative subtypes (Fig. 1D). These data demonstrate a context‐specific expression of CXCR7 in OC.

### 3.2 CXCR7 expression is strongly upregulated by estrogen in ERα‐positive ovarian cancer cells

Given the correlation with ERα expression, we found elevated ESR1 levels in both ovarian tumor stroma and epithelial compartments compared to their respective normal counterparts (Fig. 1E and Fig. S1C). We thus addressed whether CXCR7 gene was under estrogenic regulation by treating OC cells with 17β‐estradiol (E2). OVCAR‐3 cells showed a significant induction of CXCR7 expression levels in response to E2, reaching a near sevenfold increase compared with untreated cells (Fig. 1F). However, CXCR7 was not regulated by E2 in ERα‐negative TOV21G cells and in ERα‐defective SKOV‐3 cells, consistent with the requirement of a functional ERα. As a control, ERα‐positive cells exhibit a functional response to E2 with increased expression of progesterone receptor (PGR), a known target gene of ERα (Fig. S1D). These results identify CXCR7 as an estrogenic regulated gene in OC cells.

### 3.3 ERα mediates the estrogenic induction of the CXCR7 gene

**Figure 2:**
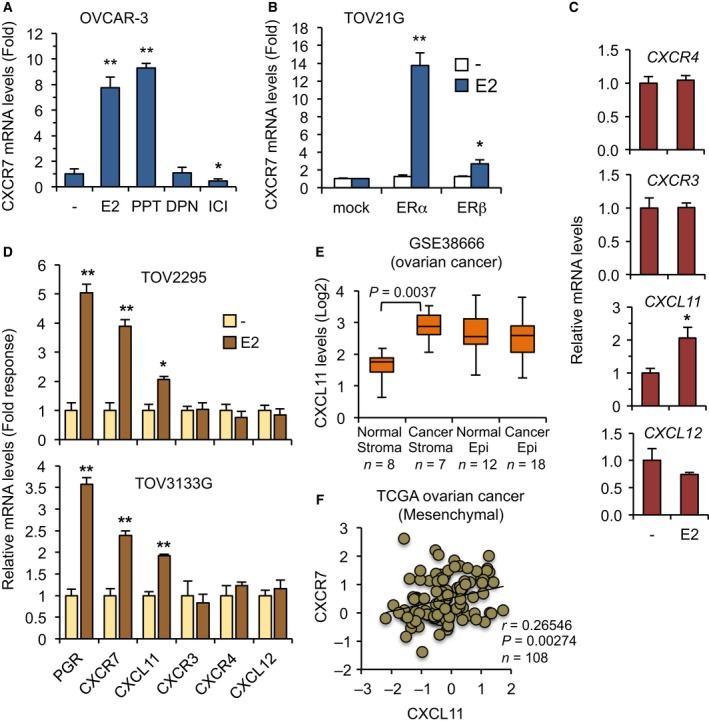
Regulation of CXCR7 expression by ERα and expression correlation of the CXCR7/CXCL11 axis in human ovarian tumors. (A) Estrogen upregulates CXCR7 gene expression through ERα in OC cells. OVCAR‐3 cells were treated with 10 nm each of E2 or selective agonist for ERα (PPT) or ERβ (DPN) for 16 h. ER antagonist ICI 182,780 was also used at 100 nm. Cells were then analyzed for CXCR7 expression by qPCR. Values represent fold response (mean ± SEM) compared with vehicle‐treated cells and are derived from at least three independent experiments performed in triplicates. Data were analyzed using Student's *t*‐test. *P < 0.03; **P < 0.001 versus vehicle‐treated cells. (B) ER‐negative parental TOV21G cells were stably transfected with human ERα or ERβ coding vector and compared to control (mock) cells. Cells were then treated with 10 nm E2 and harvested for CXCR7 expression as in A. Data are expressed as mean ± SEM, and statistical analysis was performed as in A. *P < 0.03; **P < 0.001 versus respective vehicle‐treated cells. (C) Regulation of chemokine genes by estrogen in OC cells. OVCAR‐3 cells were treated with 10 nm E2 for 16 h and gene expression determined by qPCR. Data are expressed as fold response (mean ± SEM) compared with vehicle‐treated cells, and statistical analysis was performed as in A. *P < 0.03 versus respective vehicle‐treated cells. (D) Human TOV2295 and TOV3133G cells were treated with 10 nm E2 for 16 h and gene expression determined by qPCR. Data are expressed as fold response (mean ± SEM) compared with vehicle‐treated cells set at 1.0 for each gene. Statistical analysis was performed as in A. *P < 0.03; **P < 0.001 versus respective vehicle‐treated cells. (E) Boxplots of the expression level of CXCR7 in microdissected normal and tumor samples of GSE38666. (F) Scatter plot and Pearson correlation showing the expression level of CXCR7 and that of CXCL11 in the mesenchymal subtype of the ovarian TCGA dataset. Pearson correlation score and *P* value are indicated.

Considering that OVCAR‐3 cells do express both ERα and ERβ isoforms, and that expression of the ESR2 (ERβ) gene was also found elevated in microdissected ovarian tumor stroma compared to normal sections (Fig. S2A), we thus analyzed the respective contribution of ERα and ERβ in regulating CXCR7 gene in OC cells. Using ER selective agonists able to upregulate ER target gene expression (Fig. S2B), we found that only the ERα agonist PPT, and not the ERβ agonist DPN, significantly induced CXCR7 expression in OVCAR‐3 cells (Fig. 2A). In addition, treatment of cells with the ER antagonist ICI 182,780 (also referred to as fulvestrant), which promotes ER degradation, led to a decrease in CXCR7 gene expression. To further ascertain the required role of ERα, the stable expression of ERα in ER‐negative ovarian TOV21G cells (Fig. S2C) conferred a near 14‐fold upregulation of CXCR7 in response to E2 as compared to mock‐stable control cells (Fig. 2B). In comparison with ERα, ERβ was far less effective in inducing CXCR7 expression in stable cells (Fig. 2B and Fig. S2D). These results demonstrate the predominant role of ERα in mediating the upregulation of CXCR7 expression by estrogen in OVCAR‐3 cells.

### 3.4 Components of the CXCR7/CXCL11 chemokine axis are regulated by estrogen and correlate with OC mesenchymal subtype

Chemokine receptors are known to exhibit pleiotropic and redundant responses to specific chemokine ligands, defining various and complex activation pathways. SDF‐1/CXCL12 chemokine shares interaction with CXCR4 and CXCR7 receptors, whereas I‐TAC/CXCL11 can bind to CXCR7 and CXCR3 receptors. We thus addressed whether these various chemokine components were also regulated by estrogen in OVCAR‐3 cells. I‐TAC/CXCL11 expression was found significantly induced by E2, whereas CXCR4, CXCL12, and CXCR3 remained mostly unaffected in OVCAR‐3 cells, suggesting that genes of the CXCR7/CXCL11 chemokine axis were preferably upregulated by estrogen compared to CXCR4/CXCL12 axis components (Fig. 2C). Using ERα‐positive TOV2295 and TOV3133G cells generated from human ovarian carcinomas (Letourneau *et al*., 2012), we also found a similar expression profile with the upregulation of CXCR7 and CXCL11 in response to estrogen, which further validates the distinct regulation in OC cells (Fig. 2D). As for CXCR7, CXCL11 was also significantly elevated in ovarian tumor stroma compared with normal sections (Fig. 2E), while stromal CXCR3 expression was not (Fig. S2E,F), emphasizing a selective contribution of the CXCR7/CXCL11 axis. Consistent with such positive relation, CXCR7 expression was significantly correlated with that of CXCL11 in the mesenchymal subtype of ovarian tumors (Fig. 2F). Interestingly, when performing a similar Pearson analysis using the other three OC subtypes from the TCGA dataset, no significant correlation could be found between CXCR7 and CXCL11 levels (Fig. S3A–C), implying a positive CXCR7/CXCL11 relation only in the mesenchymal cluster of OC. Given their respective regulation by estrogen, this suggests a context‐dependent regulation of the CXCR7/CXCL11 axis components in OC.

### 3.5 Identification of functional EREs and favorable active chromatin landscape at the CXCR7 locus

**Figure 3:**
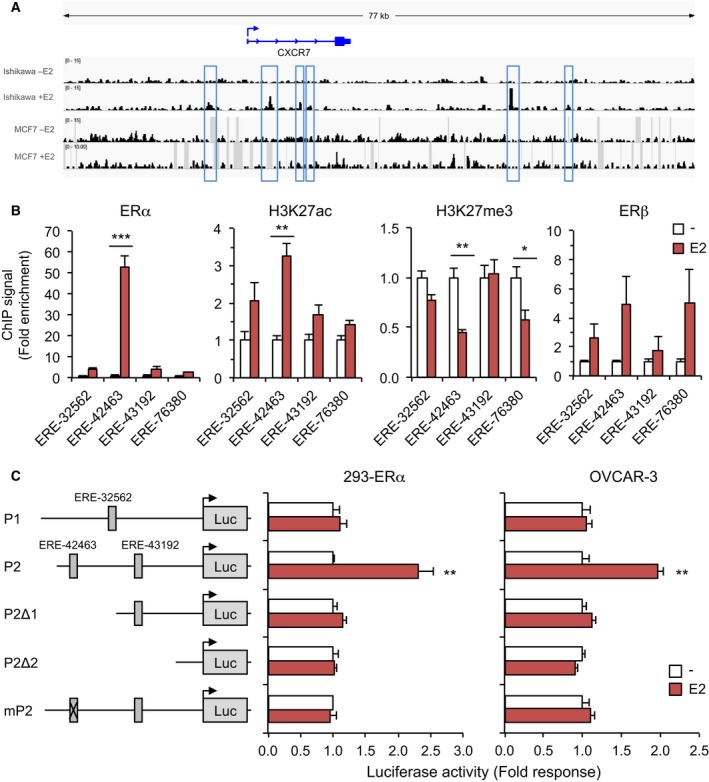
Schematic of predicted and ChIP‐validated ERα‐binding sites within the human CXCR7 locus. (A) UCSC genome view of ERα enrichment at the CXCR7 locus as determined by ChIP‐seq analysis. Peaks were mapped from Ishikawa and MCF‐7 cells treated with vehicle and 10 nm estradiol (E2), and aligned on the reference genome hg38. (B) ChIP‐qPCR assessment of ERα binding sites identified from ChIP‐seq analysis at the CXCR7 gene. OVCAR‐3 cells were treated with 10 nm E2 for 45 min and compared with vehicle treatment. ChIP‐qPCR mapping of active histone H3K27ac and repressive H3K27me3 marks, and of ERβ, relative to each ERE was also performed. ChIPed proteins are indicated on top of each graph. Values (mean ± SEM) were derived from at least three independent ChIP experiments performed in duplicates and analyzed using Student's *t*‐test. *P < 0.05; **P < 0.01; ***P < 0.001 versus vehicle‐treated cells. (C) Activation of human CXCR7 proximal P2 promoter by E2. Luciferase reporter gene constructs were prepared using DNA fragments corresponding to proximal P1 (upstream from the transcriptional start site) and P2 (in intron 1) promoter regions of CXCR7 gene containing the indicated EREs as schematized. Luciferase assay was performed in 293 cells transfected with ERα (left) and in OVCAR‐3 cells (right) treated or not with 10 nm E2 for 16 h. Truncated P2Δ1 and P2Δ2 fragments, and ERE‐42463 mutated P2 (mP2) were also analyzed. Luciferase activities were normalized to β‐galactosidase activity and expressed as fold response (mean ± SEM) compared with untreated cells set at 1.0 for each construct. Data were analyzed using Student's *t*‐test. **P < 0.01 versus vehicle‐treated cells.

Estrogen responsiveness of target genes is mostly mediated through binding of ERs to ERE. Given the prominent role of ERα in CXCR7 upregulation, we then performed analysis using our ERα ChIP‐seq data (Edjekouane *et al*., 2016) in comparison with available data obtained in Ishikawa cells (Encode) and sequence screening of consensus EREs, to identify putative ERα binding sites in the vicinity of the CXCR7 gene. Sequence analysis has revealed several putative EREs within the CXCR7 gene and also at more distal locations, among which ERE‐32562 resides upstream of 5′‐UTR containing exon 1 in the proximal promoter region of CXCR7, and ERE‐42463 and ERE‐43192 are located in the first intron upstream of the coding exon 2 (Fig. 3A). Standard ChIP validation revealed that the ERE‐42463 was by far the most potent in recruiting ERα with a more than 52‐fold increase in OVCAR‐3 cells treated with E2 compared to control, whereas a threefold to fivefold response was observed for the other EREs tested (Fig. 3B). To further distinguish between active and inactive EREs, we performed ChIP for transcriptionally active histone H3K27ac marks and inactive H3K27me3 marks at each respective ERE. We found a strong enrichment of H3K27ac mark in response to E2 at the ERE‐42463, whereas the H3K27me3 mark was reduced (Fig. 3B), suggesting that estrogen induces a favorable environment for active transcription at the ERE‐42463 in OC cells. Such enrichment of H3K27ac mark is also indicative of estrogenic regulation of the PGR promoter (Fig. S4). In contrast, our ChIP‐seq data indicate ERE‐42463 as a very weak ERα recruiting site in MCF‐7 cells (Fig. 3A), supporting a selective and context‐dependent role of the ERE‐42463 in OC cells. As for the other EREs tested, lower levels of active chromatin marks were found in response to E2, although a reduction in H3K27me3 mark was observed at the ERE‐76380, suggesting a less active but functional ERE to mediate distal estrogenic regulation of CXCR7 (Fig. 3A,B). Although highly variable, ERβ was less recruited to the ERE‐42463 compared with ERα (Fig. 3B), supporting an ER isoform‐specific preference of recruitment. These results demonstrate a direct role of ERα in interacting with CXCR7 promoter in response to estrogen, which triggers an active chromatin landscape as part of the mechanism responsible to enhance CXCR7 expression in OC cells.

We next addressed the functional role of CXCR7 EREs in mediating E2‐induced transcription using luciferase assay. We observed that the region flanking exon 1 of CXCR7 (hereby termed P1 promoter) was not responsive to estrogen in ERα‐expressing 293 cells and OVCAR‐3 cells, consistent with the inactivity of ERE‐32562 (Fig. 3C). However, the region of intron 1 (termed P2), which contains ERE‐42463 and ERE‐43192, was highly responsive to estrogen in both cell types. In addition, truncation analysis and site‐directed mutagenesis revealed that the ERE‐42463 was required to promote estrogenic response of the P2 promoter region (Fig. 3C). These data identify a functional ERE in the P2 promoter of the CXCR7 gene, which is critical to mediate estrogen‐induced transcriptional upregulation of CXCR7 expression in OC cells.

### 3.6 CXCR7 activation enhances the transcriptional activity of ERα

**Figure 4:**
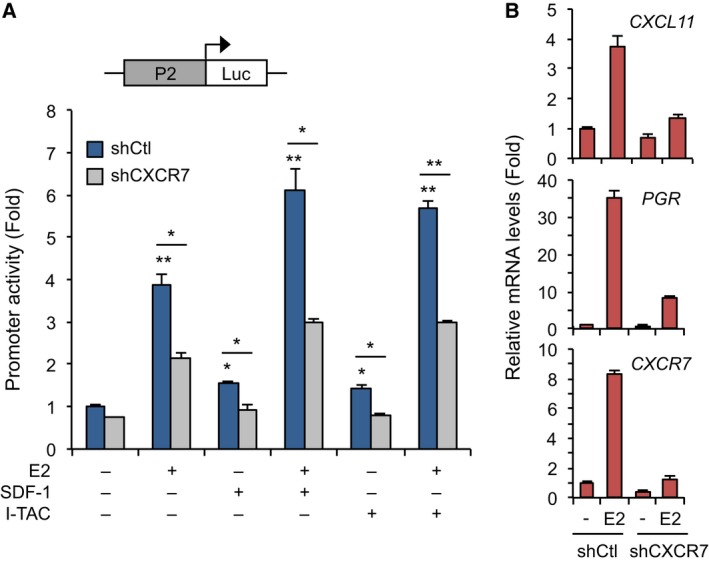
CXCR7 is required for maximal induction of ERα activity. (A) Cross‐regulation of CXCR7 P2 promoter activity with ERα. shCXCR7‐infected OVCAR‐3 cells were transfected with P2‐Luc reporter and subjected to luciferase assay compared to control shCtl cells. Cells were treated or not with 10 nm E2, 25 nm I‐TAC, or 25 nm SDF‐1 for 16 h. Results were normalized to β‐galactosidase activity and expressed as fold response compared with untreated shCtl cells set at 1.0. Values (mean ± SEM) were derived from at least three independent experiments performed in triplicates and analyzed using Student's *t*‐test. *P < 0.01; **P < 0.001. (B) CXCR7 is required for maximal induction of estrogen‐responsive genes. qPCR analysis of PGR and CXCL11 expression in shCtl and shCXCR7‐expressing stable OVCAR‐3 cells treated or not with 10 nm E2 for 16 h. Each expression value was quantified to RPLP0 and expressed as fold response compared with untreated respective cells. Results were derived from at least three independent ChIP experiments performed in triplicates. Bars represent SEM.

In line with the upregulation of CXCR7 and CXCL11 by ERα, we next determined whether CXCR7 activation and signaling could regulate ER transcriptional response in a feedback control fashion. We found that the estrogen‐induced activation of ERα in OVCAR‐3 cells was better potentiated in the presence of CXCR7 agonists SDF‐1 and I‐TAC, supporting a role of CXCR7 activation on ERE‐driven P2 reporter activity (Fig. 4A). Consistent with this, CXCR7 knockdown strongly impaired the response to SDF‐1 and I‐TAC, but also that of estradiol on ERα activity, indicating the requirement of CXCR7 to achieve an optimal response of the P2 promoter to estrogen. In addition, ectopic expression of CXCR7 in 293 cells also potentiated ERα activation by estradiol, which was further enhanced with SDF‐1 treatment (Fig. S5). Such enhancement of estrogenic response by CXCR7 also had an impact on target gene expression with significant impairment in the increase in PGR and CXCL11 gene expression to estrogen in conditions of CXCR7 depletion (Fig. 4B). These results suggest that the CXCR7 activation pathway contributes to ERα transcriptional competence in OC cells.

### 3.7 Ser‐118 phosphorylation mediates ERα response to CXCR7 activation

**Figure 5:**
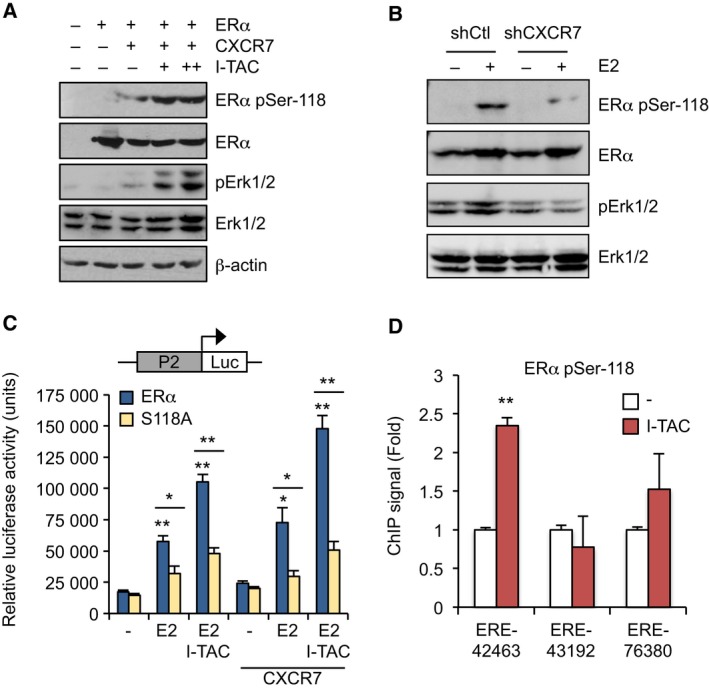
CXCR7 signaling triggers ERα Ser‐118 phosphorylation and chromatin recruitment. (A) CXCR7 activation enhances ERα phosphorylation at Ser‐118; 293 cells were transfected with ERα and CXCR7 as indicated and then treated or not with 25 and 50 nm I‐TAC for 30min. Western analysis was performed using pan and pSer‐118‐specific ERα antibodies. Erk1/2 phosphorylation was also monitored in samples, and β‐actin for control loading. (B) Estrogen‐induced Ser‐118 phosphorylation is dependent on CXCR7. Stable shCtl and shCXCR7‐expressing OVCAR‐3 cells were treated or not with 10 nm E2 and then subjected to Western analysis as in A; (C) 293 cells were transfected with wild‐type or S118A mutated ERα in presence of P2‐Luc reporter, and then treated or not with 10 nm E2 and 25 nm I‐TAC for 16 h. Luciferase values were normalized to β‐galactosidase activity and expressed as fold response compared with respective untreated cells set at 1.0. Values (mean ± SEM) were derived from at least three independent ChIP experiments performed in triplicates and analyzed using Student's *t*‐test. *P < 0.01; **P < 0.001. (D) I‐TAC promotes recruitment of Ser‐118 phosphorylated ERα to the CXCR7 gene. OVCAR‐3 cells were treated with 50 nm I‐TAC for 45 min and subjected to ChIP‐qPCR analysis of ERα binding sites identified from ChIP‐seq analysis. An antibody against ERα pSer‐118 was used for ChIP. Values (mean ± SEM) were derived from at least three independent ChIP experiments performed in duplicates and analyzed using Student's *t*‐test. **P < 0.001 versus respective vehicle‐treated cells.

To determine how CXCR7 can modulate ERα activity, we tested the possibility that CXCR7 signaling might promote ERα phosphorylation. Phosphorylation of ERα is known to directly affect its transcriptional potential with ERα Ser‐118 being considered a major site of phosphorylation in response to estrogen and Erk activation (Sanchez *et al*., 2010; Weigel and Moore, 2007). We found that phosphorylation of ERα at Ser‐118 was increased in response to CXCR7 activation with I‐TAC, concomitant with increased Erk1/2 activation in 293 cells (Fig. 5A). Also, the phosphorylation of Ser‐118 normally triggered by E2 was strongly impaired by CXCR7 knockdown in OVCAR‐3 cells (Fig. 5B). Maximal ERα transcriptional response to CXCR7 activation also required Ser‐118, as the S118A mutation significantly reduced ERα transcriptional response to I‐TAC (Fig. 5C). In addition, I‐TAC potently increased the enrichment of phosphorylated Ser‐118 ERα at the ERE‐42463 of the CXCR7 P2 promoter in ChIP assay, supporting a mechanism of ERα recruitment to upregulate CXCR7 expression in OVCAR‐3 cells (Fig. 5D). These findings identify Ser‐118 as a targeted site of CXCR7 signaling pathway, resulting in ERα phosphorylation and activation that contributes to enhancing CXCR7 expression in a positive feedback manner.

### 3.8 CXCR7 and CXCL11 positively correlate with the mesenchymal pattern in ovarian tumors

**Figure 6:**
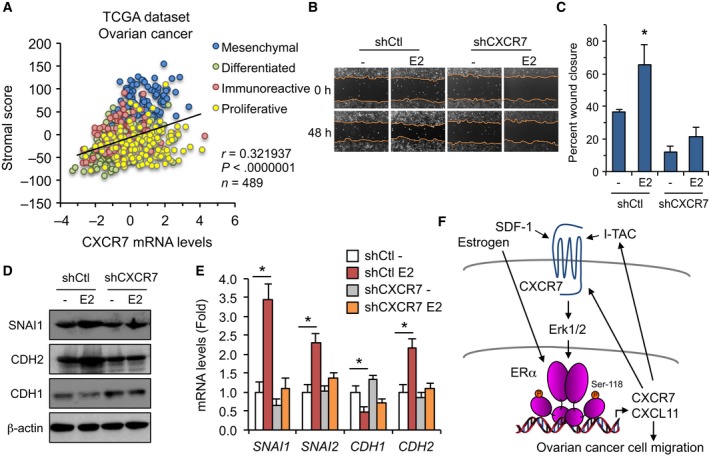
The ERα‐CXCR7 cross‐regulation induces the EMT pathway and migration of OC cells. (A) Scatter plots of CXCR7 expression compared with the stromal signature score for each subtype in the ovarian TCGA dataset. Pearson correlation rate and *P* value are indicated. (B) Representative images of linear wounds made on shCXCR7 stably expressing OVCAR‐3 cells compared with shCtl control cells. Cells were treated or not (vehicle) with 10 nm E2 over a period of 48 h. (C) Quantitative determination of wound closure calculated as % wound area healed relative to 0‐h time period. Results were recorded from three independent experiments performed in duplicate. Data were analyzed using Student's *t*‐test. Bars represent SEM. *P < 0.05 versus control vehicle‐treated cells. (D) Estrogen induction of the EMT pathway is dependent on CXCR7. shCXCR7‐expressing OVCAR‐3 cells and control shCtl cells were treated with 10 nm E2 or vehicle for 16 h. Western analysis was performed on EMT markers and β‐actin used for control loading. (E) qPCR analysis was performed on shCXCR7‐expressing OVCAR‐3 cells treated as in (D) and compared with control shCtl cells. Expression levels of EMT genes were normalized to RPLP0 and expressed as fold response compared with untreated cells. Results were derived from three independent experiments performed in triplicate. Data were analyzed using Student's *t*‐test. Bars represent SEM. *P < 0.05 versus control vehicle‐treated cells. (F) Proposed model of ERα‐CXCR7 interplay in OC cells. Estrogenic activation of ERα results in increased expression of CXCR7, identified as a direct target gene, and of CXCL11/I‐TAC gene, which in turn triggers the Erk pathway that promotes Ser‐118 phosphorylation and feedback ERα activation. Although not regulated by estrogen, SDF‐1 also a CXCR7 ligand can as well promote CXCR7 signaling to activate ERα. Such interplay determines an ERα‐CXCR7 feed‐forward mechanism that results in activation of the EMT pathway and enhanced migration potential of OC cells.

Our gene set enrichment analysis (GSEA) of microdissected profiles and TCGA variant subtypes revealed an enriched expression of CXCR7 in the stromal compartment of ovarian tumors. We have defined the correlation analysis of CXCR7 gene expression against a gene stromal signature that was built from overlapping genes of mesenchymal markers and ECM remodeling derived from the TCGA mesenchymal subtype and the Tothill C1 (tumor desmoplasia) datasets (Verhaak *et al*., 2013; Yang *et al*., 2017). The signature was discriminately expressed in the different OC subtypes with a significant enrichment in the mesenchymal subtype (Fig. S6A). When compared to CXCR7 expression, a positive correlation with the signature score was found using Pearson analysis in all OC subtypes of the TCGA dataset (*r* = 0.32; Fig. 6A). Supporting this, CXCR7 levels positively correlated with classic ECM and reactive stromal cell markers (Fig. S6B). CXCL11 levels were also correlated with the stroma score although to a lesser extent (*r* = 0.14), largely owing to the fact that the only significant rates were observed in the mesenchymal and differentiated subtypes (Fig. S6C), thereby supporting a more context‐dependent effect for CXCL11. To further validate their possible contribution to OC mesenchymal subtype, we found that both CXCR7 and CXCL11 strongly correlate with genes associated with ECM and OC invasion, such as PRRX1, TMEM45A, and CTSK, when analyzed in the mesenchymal counterpart of ovarian tumors (Fig. S7). This suggests a prominent role of the CXCR7/CXCL11 axis in the mesenchymal subtype which strongly correlates with the stromal characteristics of tumors.

### 3.9 CXCR7 is required to increase the migration potential of ovarian cancer cells to estrogen

The positive relation of CXCR7 with several components of reactive stromal cells and invasive tumor cells does suggest a role for CXCR7 to affect OC cell migration. We thus addressed whether CXCR7 regulation of estrogenic response exerts a functional impact on OC cell mobility. An increased migration of OVCAR‐3 cells was observed in response to estrogen when compared to untreated cells, reaching a near 70% of wound closure over 48 h of treatment (Fig. 6B,C). However, CXCR7 depletion strongly impaired the estrogenic potential of cells to migrate, suggesting that CXCR7 was required to promote maximal migration of OVCAR‐3 cells. To support these findings, we analyzed the expression of classic EMT marker genes, such as the mesenchymal markers CDH2/N‐cadherin, SNAI1/Snail, and SNAI2/Slug. Expression levels of these EMT genes were found positively correlated with CXCR7 in the whole TCGA dataset of OC subtypes (Fig. S8), implying a critical role of CXCR7 in mesenchymal transition phenotype. When treating OVCAR‐3 cells, we found that estrogen markedly increased SNAI1/Snail protein and mRNA expression, with a concomitant reduction in CDH1 (Fig. 6D,E). These findings are consistent with the repressive effect of Snail transcription factor on CDH1/E‐cadherin epithelial marker gene and support a transition toward a metastatic phenotype. Likewise, SNAI2/Slug and CDH2/N‐cadherin expression levels were also increased by estrogen in these conditions. However, CXCR7 knockdown in OVCAR‐3 cells mostly abolished the upregulation of SNAI1, SNAI2, and CDH2 expression by estrogen (Fig. 6D,E). These results indicate that CXCR7 expression is required to promote a mesenchymal transition phenotype to OVCAR‐3 cells when exposed to estrogen, and support a feedback regulation of CXCR7 to activate the EMT pathway in OC cells.

## 4 Discussion

Ovarian cancer is certainly one of the most intractable diseases, resulting in poor survival rates as most patients are asymptomatic until the disease has metastasized. Despite tremendous outcomes in our understanding of ovarian tumorigenesis, the exact mechanistic events that drive metastasis of ovarian tumor cells have not yet been well elucidated. Estrogen is a well‐known risk factor for OC progression and spread, but little is known about its contribution at the molecular level. This study identifies a mechanism by which CXCR7/ACKR3 gene is activated by estrogen, through the direct recruitment of ERα and favorable chromatin modifications in OC cells. Estrogen also induces CXCL11 gene expression, resulting in a feedback regulation of ERα activation and CXCR7 gene transcription. This ERα‐CXCR7 cross‐regulation helps to promote an optimal response to estrogen and migration potential in OC cells.

Recent studies have implicated CXCR7 overexpression with tumor aggressiveness and poor prognosis in a number of cancers, such as breast, prostate, and lung cancer (Iwakiri *et al*., 2009; Miao *et al*., 2007; Wang *et al*., 2008; Wani *et al*., 2014). However, the mechanism underlying such regulation of CXCR7 expression remains unclear. VEGF was shown to upregulate CXCR7 in human hepatocellular carcinoma cells (Zheng *et al*., 2010), and IL‐8 induced CXCR7 expression in prostate cancer cells (Singh and Lokeshwar, 2011). Although reports on the role of CXCR7 in OC are more limited, activation of CXCR7 by SDF‐1 was recently shown to promote OC cell invasion (Yu *et al*., 2014), whereas CXCR7 expression levels were found similar in normal and neoplastic ovaries (Jaszczynska‐Nowinka *et al*., 2014). Our data identify estrogen as an upstream signal and potent inducer of CXCR7 expression, through direct binding of ERα to the CXCR7 proximal promoter, thereby inducing favorable chromatin activation and CXCR7 gene transcription. This identifies CXCR7 as an ERα target gene in OC cells. Moreover, the increase also observed in CXCL11 expression defines the CXCR7/CXCL11 chemokine axis as an important pathway of OC cell invasion regulated by estrogen. Estrogen replacement therapy is a strong risk factor for OC in postmenopausal women, often resulting in aggressive dissemination and chemoresistance (Beral *et al*., 2007; Kommoss *et al*., 2016; Zhou *et al*., 2008). Our findings are consistent with such effect of exogenous estrogen and provide a mechanistic understanding of how cancer cells may exhibit metastatic behavior triggered by the CXCR7 activation axis.

Redundancy in chemokine actions has complicated our understanding of specific pathways involved in tumorigenesis. Overexpression of the SDF‐1/CXCL12 chemokine and its most recognized receptor CXCR4 has been associated with the progression phenotype of several epithelial cancers, including OC (Figueras *et al*., 2018; Guo *et al*., 2014; Popple *et al*., 2012). Besides interacting with SDF‐1, CXCR7 activation by I‐TAC is adding an additional layer of control to cell growth and adhesion properties (Balabanian *et al*., 2005; Burns *et al*., 2006; Miao *et al*., 2007; Wang *et al*., 2008). Here, we describe a selective increase in CXCR7 and CXCL11 gene expression in estrogen‐treated OC cells, compared to CXCR4, CXCL12, and CXCR3 genes, which remained largely unaffected. This suggests a primary role of the CXCR7/CXCL11 chemokine axis in the response of OC cells to estrogen, bringing selectivity in targeting the CXCR7 axis compared to other ER‐positive gynecologic cancer cells, such as breast cancer cells which did not exhibit CXCR7 upregulation by estrogen (Boudot *et al*., 2011). This specific regulation of CXCR7 and CXCL11 also correlates with data mining analysis, revealing enhanced expression levels of both genes in microdissected sections of OC stromal compartments as well as significant positive correlation rates in OC mesenchymal subtype. In comparison, CXCR3 gene was not upregulated in human OC tumor subtypes and no significant changes were observed in response to estrogen in OC cells, implying a positive effect mainly associated with the CXCR7/CXCL11 axis. Given the upregulation of CXCR7 and CXCL11 expression by estrogen, our results suggest that exposure of OC cells to estrogen might promote a favorable transition to transcriptional activation possibly of both genes, most likely by inducing preferred recruitment of ligand‐bound ERα and modifications of chromatin marks, such as enrichment of H3K27ac and reduction in H3K27me3 marks. Although direct ERα binding and activating histone changes are shown to take place for CXCR7 gene upregulation by estrogen, it remains to be determined whether such modifications of promoter context also apply to mediate the estrogenic induction of the CXCL11 gene, given its positive response and enhanced expression.

Mostly based on studies in breast cancer cells, site‐specific phosphorylation is known to regulate ERα transcriptional competence (Sanchez *et al*., 2010; Weigel and Moore, 2007). Ser‐118 is a major phosphorylation site implicated in the activation of ERα, and clinical correlations have been established with resistance and survival of patients with breast cancer (Chen *et al*., 2013; Kok *et al*., 2009; Yamashita *et al*., 2008). Our findings indicate that Ser‐118 is also a targeted site for ERα phosphorylation in OC cells. Although the role of phosphorylated ERα in ovarian function and tumorigenesis is unknown, we identify CXCR7 as a signaling inducer of ERα Ser‐118 phosphorylation through activation of Erk, leading to enhanced ERα transcriptional response to I‐TAC ligand and recruitment at the CXCR7 promoter. With the increased expression of CXCR7 and CXCL11 in estrogen‐treated cells, this suggests that Ser‐118 phosphorylation is involved in a feed‐forward mechanism to activate ERα and provide feedback regulation to the CXCR7/CXCL11 activation axis in OC cells. ERβ isoform is known to undergo phosphorylation in response to CXCR4 activation, resulting in activation in breast cancer cells (Sanchez *et al*., 2010; Sauve *et al*., 2009). However, such response of ERβ does not seem to occur in OC cells, as suggested by the limited action of ERβ to regulate CXCR7, its poor recruitment to CXCR7 gene, and its lack of correlation with CXCL11 in high serous ovarian tumors. Also consistent with this, a reduced expression of ERβ has been reported in OC metastatic tumors (Bossard *et al*., 2012; Kyriakidis and Papaioannidou, 2016).

Acquisition of mesenchymal features is observed in late‐stage ovarian tumors, resulting in metastatic dissemination within the peritoneal cavity and poor clinical outcome. In particular, clinical studies have demonstrated that the EMT pathway, outlined by an increase in expression of SNAI1/Snail and SNAI2/Slug, two transcription factors that repress CDH1/E‐cadherin gene, is associated with lower overall survival of patients with OC (Gallo *et al*., 2010). In fact, in contrast to most carcinomas that dedifferentiate during neoplastic progression, epithelial ovarian carcinomas retain E‐cadherin expression, thereby losing their stromal characteristics and promoting tumor progression at an early stage (Hudson *et al*., 2008). It is the subsequent reacquisition of mesenchymal features with increased Snail and Slug and reduced E‐cadherin that transforms primary lesions to late‐stage tumors with peritoneal metastatic dissemination and shorter overall survival (Gallo *et al*., 2010; Takai *et al*., 2014). Our results indicate that such gene pattern can be triggered by estrogen in OC cells, with an upregulation of SNAI1/Snail and SNAI2/Slug expression, and downregulation of CDH1 gene, implying a switch to the mesenchymal phenotype. This is also consistent with the described effect of estrogen to induce metastatic potential of OC cells (Jeon *et al*., 2016; Park *et al*., 2008). Similarly, expression of CDH2/N‐cadherin, also a mesenchymal marker, was increased in estrogen‐treated OC cells. Interestingly, these changes of EMT markers were mostly dependent on CXCR7, as depletion of CXCR7 strongly impaired their responses to estrogen. This suggests a key role for CXCR7 in mediating the estrogenic regulation of mesenchymal markers, thereby promoting a mesenchymal phenotype and invasion of OC cells. This is also consistent with the increased expression of CXCR7 and the strong correlation with CXCL11 preferably taking place in the mesenchymal subtype of ovarian tumors, providing an activation pathway for EM transition. Recent studies in breast cancer cells have revealed that CXCR7 activation by SDF‐1 increases components regulating cell adhesion and cell–matrix interaction, such as vascular adhesion molecule VCAM‐1 and matrix metalloproteinases MMP‐2 and MMP‐9 (Wani *et al*., 2014), and SDF‐1 also promoted MMP‐9 expression in OC cells (Yu *et al*., 2014). Whether CXCR7 activation with I‐TAC results in a similar effect is not known, but the requisite implication of CXCR7 in promoting EMT transition and cell motility is strongly supportive.

The significant increase in CXCR7 gene expression in the mesenchymal subtype of the ovarian TCGA dataset is highly indicative of a role of CXCR7 in the acquisition of mesenchymal features. Consistent with this, we found a positive correlation of CXCR7 expression with a gene stromal signature built from overlapping genes from the TCGA mesenchymal subtype and the Tothill C1 (tumor desmoplasia) subtype samples that were positively correlated with mesenchymal markers and ECM remodeling (Verhaak *et al*., 2013; Yang *et al*., 2017). Our GSEA of microdissected profiles revealed an enriched expression of CXCR7 in the stromal compartment of ovarian tumors in several datasets. Supporting this, CXCR7 levels positively correlated with classic ECM and reactive stromal cell markers, including FBN1 which is strongly associated with OC tumor staging and poor overall survival (Sun *et al*., 2017). CXCL11 levels were also correlated with the stroma signature in the mesenchymal and differentiated subtypes, supporting a more context‐dependent role of CXCL11. In line with such role, CXCL11 has recently been identified as a predictive marker for OC clinical outcome (Jin *et al*., 2018). In addition, when analyzed in the mesenchymal subtype, both CXCR7 and CXCL11 strongly correlate with genes associated with ECM and OC invasion phenotype. For instance, PRRX1 is considered a master regulator of a network hub driving OC metastasis (Khirade *et al*., 2015). Therefore, the stromal signature of the CXCR7/CXCL11 pathway might contribute to enhance reactive stroma profile and stromal stiffness of tumors, both being highly predictive of primary chemoresistance and poor outcome (Ryner *et al*., 2015). Such stromal expression pattern of CXCR7 and CXCL11 genes and their enrichment to mesenchymal OC subtype associated with worst prognosis support their contribution in the ability of the microenvironment to modulate cancer development, resulting in tumor dissemination.

## 5 Conclusions

Our study identifies the CXCR7/CXCL11 axis as a critical component of OC tumor stroma activation with a role of estrogen in promoting such transition, thereby determining the acquisition of metastatic phenotype and the fate of patients with OC. Therefore, as depicted in Fig. 6F, our findings suggest that in the context of estrogen, increased expression of CXCR7 and of CXCL11 under ERα control might promote a feed‐forward mechanism to induce ECM remodeling, EMT pathway, and metastatic behavior of OC cells, by maintaining supported activation of the CXCR7/CXCL11 chemokine axis. Therefore, this forward mechanism might participate in the complex gene profile heterogeneity of OC that dictates changes in tumor microenvironment and metastatic acquisition.

## Author contributions

SB, EC, and AT designed research; SB and LE performed experiments; SB, LE, KS, and EC contributed and prepared samples, reagents, and analytic tools; SB and AT analyzed data; SB, EC, and AT wrote the manuscript.

## Supporting information


**Fig. S1.** ERα and CXCR7 expression in reproductive cancer cells and tumors.
**Fig. S2.** ERβ and CXCR3 expression in ovarian cancer cells and tumors.
**Fig. S3.** Correlation analysis of CXCR7 and CXCL11 in ovarian cancer subtypes.
**Fig. S4.** Enrichment of ERα at the ERE of the PGR gene.
**Fig. S5.** Maximal activation of ERα by the CXCR7/SDF‐1 chemokine axis requires Ser‐118.
**Fig. S6.** CXCR7 and CXCL11 expression is associated with OC stromal signature and markers.
**Fig. S7.** CXCR7 and CXCL11 expression correlates with ECM markers.
**Fig. S8.** CXCR7 expression is associated with EMT indicators.
