# Supplementary material for: Feedback control of the CXCR7/CXCL11 chemokine axis by estrogen receptor α in ovarian cancer
Source: Mol Oncol. 2018 Aug 23;12(10):1689–705. doi: 10.1002/1878-0261.12362 (PMC6165996; doi:10.1002/1878-0261.12362)
Supplement: Supplementary file 1 — Fig. S1. ERα and CXCR7 expression in reproductive cancer cells and tumors. Fig. S2. ERβ and CXCR3 expression in ovarian cancer cells and tumors. Fig. S3. Correlation analysis of CXCR7 and CXCL11 in ovarian cancer subtypes. Fig. S4. Enrichment of ERα at the ERE of the PGR gene. Fig. S5. Maximal activation of ERα by the CXCR7/SDF‐1 chemokine axis requires Ser‐118. Fig. S6. CXCR7 and CXCL11 expression is associated with OC stromal signature and markers. Fig. S7. CXCR7 and CXCL11 expression correlates with ECM markers. Fig. S8. CXCR7 expression is associated with EMT indicators. [file MOL2-12-1689-s001.pdf]

Fig. S1

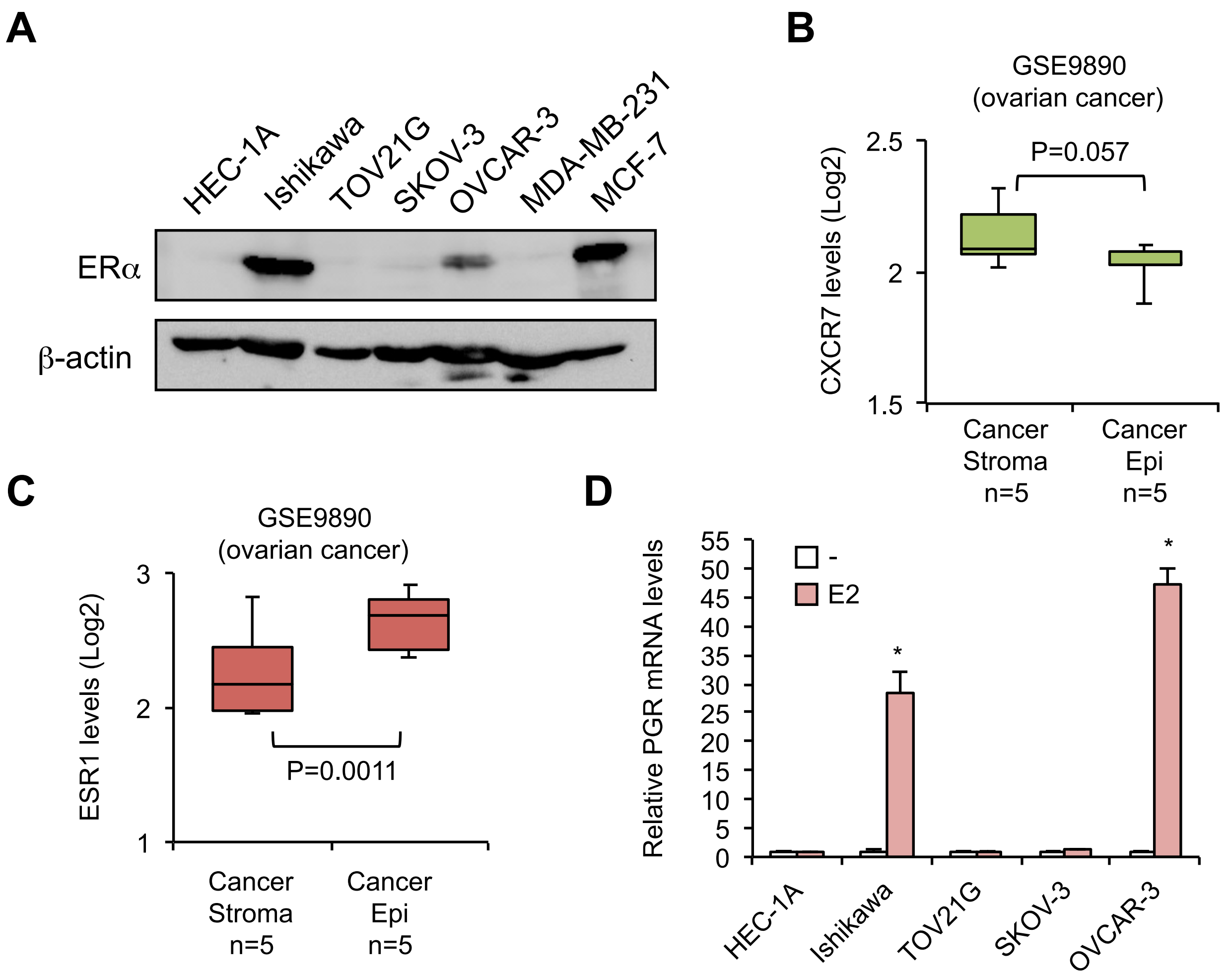


**Fig. S1. ERα and CXCR7 expression in reproductive cancer cells and tumors**

**A-** ERα protein levels in endometrial, ovarian and breast cancer cells determined by Western analysis. **B-** Boxplots of the expression levels of CXCR7 in the microdissected tumor stroma and epithelium of GSE9890 dataset. **C-** Boxplots of the expression levels of ESR1 in the GSE9890 dataset. **D-** qPCR analysis of progesterone receptor (PGR) gene expression in cells treated with 10nM estradiol (E2) for 16hrs. Results were normalized to RPLP0 expression. Values represent mean±SEM derived from three independent experiments performed in triplicate. Data were analyzed using Student's t-test. *, P < 0.001 versus control vehicle-treated cells.

# Fig. S2

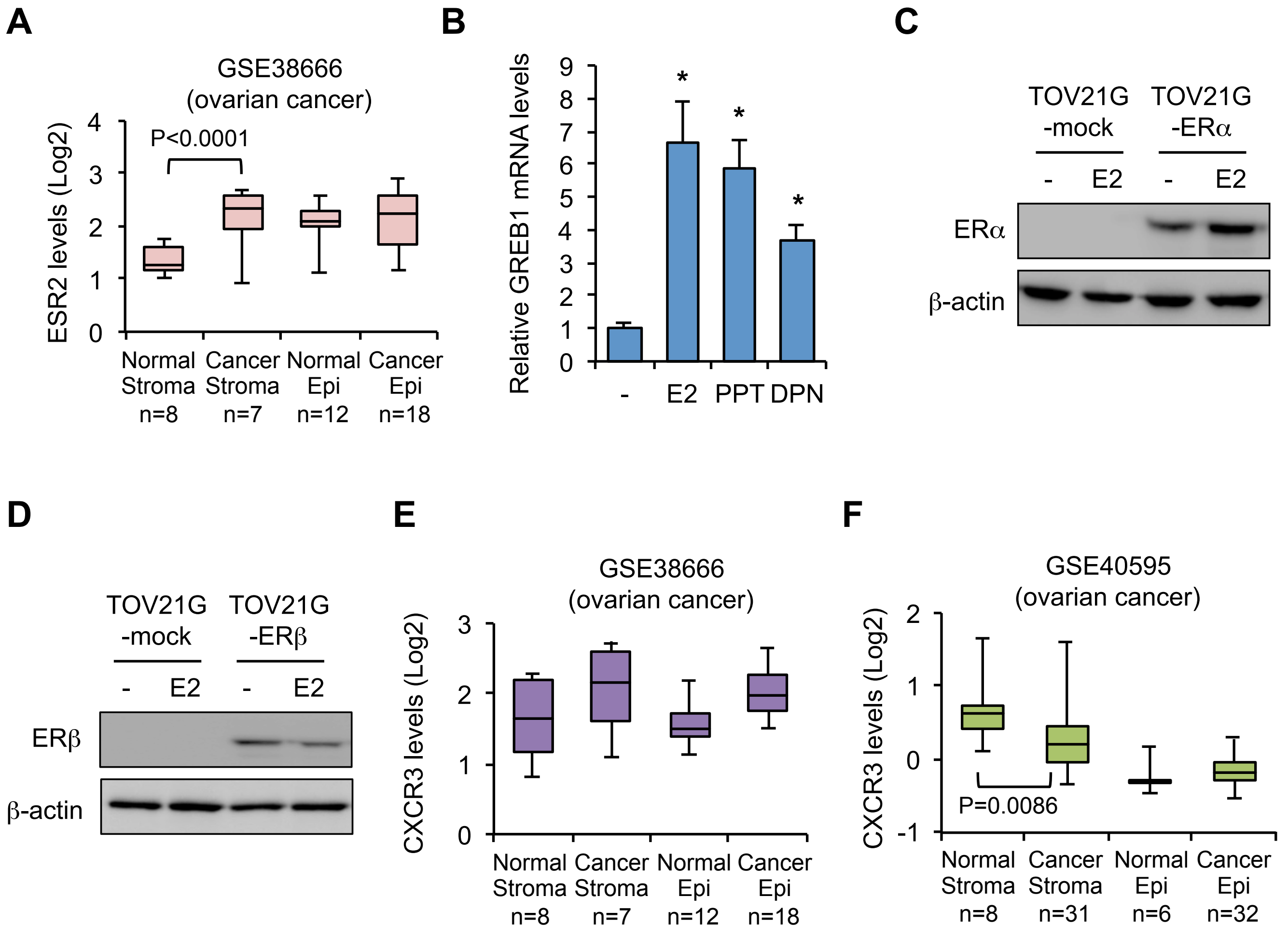


**Fig. S2. ERβ and CXCR3 expression in ovarian cancer cells and tumors**
**A-** Boxplots of the expression levels of ESR2 (ERβ) in microdissected normal epithelium, normal stroma, tumor epithelium and tumor stroma included in the ovarian dataset GSE38666. **B-** OVCAR-3 cells were treated with 10 nM each of E2 or selective agonist for ERα (PPT) or ERβ (DPN) for 16 hrs. Cells were then analyzed for GREB1 expression by qPCR. Values represent fold response compared to vehicle-treated cells. Results were derived from three independent experiments performed in triplicate. Errors represent SEM. Data were analyzed using Student's t-test. *, $P < 0.005$ versus control vehicle-treated cells. **C-** ER-negative ovarian cancer TOV21G cells were stably transfected with human ERα. Validation of expression was performed by Western analysis of cells treated or not with 10nM E2 and compared to mock-transfected parental cells. **D-** Similar as in (C) except that TOV21G cells were stably transfected with ERβ. **E-** Boxplots of the expression levels of CXCR3 in the GSE38666 dataset. **F-** Boxplots of the expression levels of CXCR3 in the GSE40595 dataset.

## Fig. S3

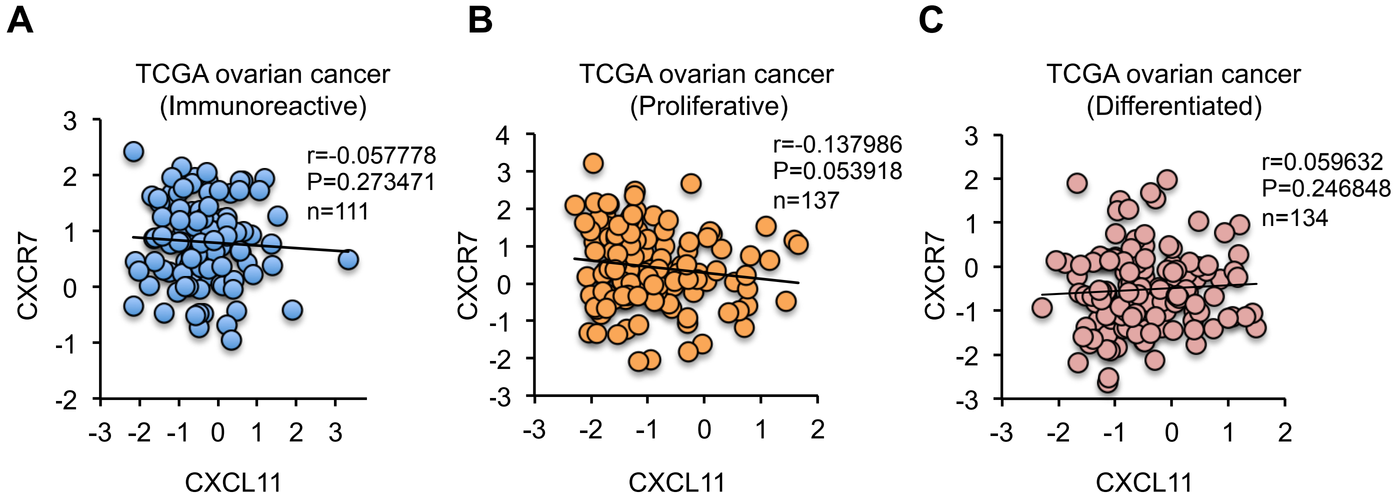


**Fig. S3. Correlation analysis of CXCR7 and CXCL11 in ovarian cancer subtypes.**
**A-C** Scatter plots of CXCR7 and CXCL11 expression levels in the immunoreactive **(A)**, proliferative **(B)**, and differentiated **(C)** molecular subtypes stratified from the ovarian TCGA dataset. Pearson correlation scores (r) and P values are indicated for each.

**Fig. S4**

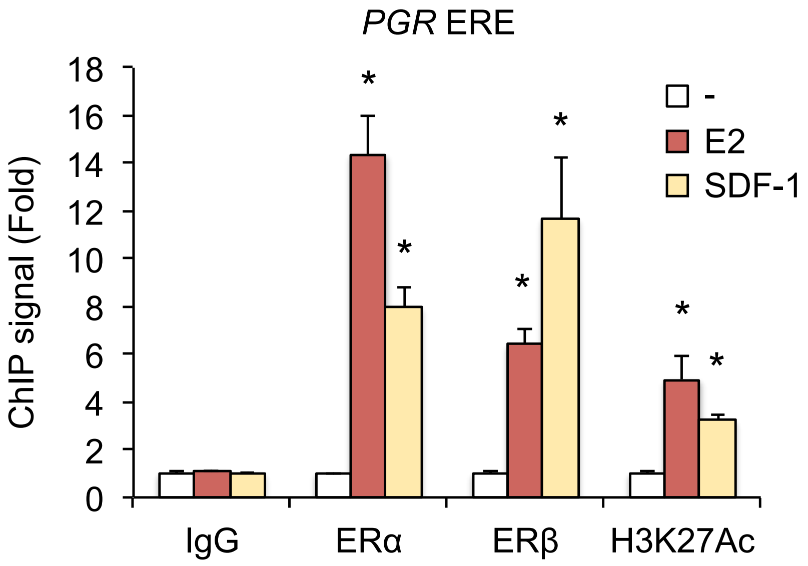


**Fig. S4. Enrichment of ERα at the ERE of the PGR gene**
OVCAR-3 cells were treated or not with 10nM E2 or 25nM SDF-1 for 45 min and harvested for ChIP-qPCR analysis using antibodies for ERα, ERβ, or active histone H3K27ac mark. Preimmune IgGs were used as a negative control. Values represent mean±SEM derived from three independent ChIP experiments performed in duplicate. Data were analyzed using Student's t-test. *, $P < 0.01$ versus control vehicle-treated cells.

**Fig. S5**

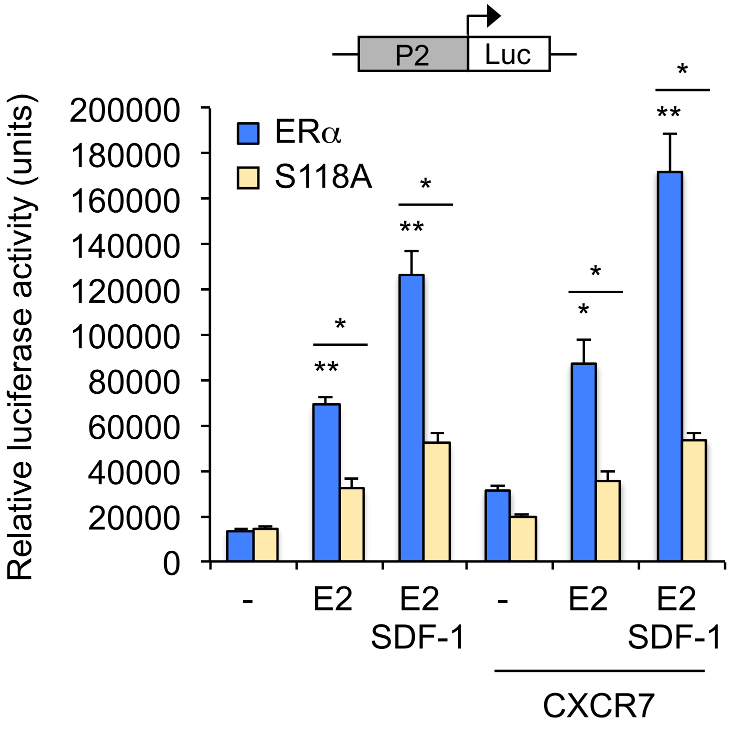


**Fig. S5. Maximal activation of ERα by the CXCR7/SDF-1 chemokine axis requires Ser-118**

293 cells were transfected with wild-type ERα or S118A mutant in the presence of a luciferase reporter gene under the control of CXCR7 P2 promoter (P2-Luc). Cells were also transfected with CXCR7 as indicated. Cells were then treated or not with 10nM E2 and 50nM SDF-1 for 16h. Luciferase values were normalized to β-galactosidase activity and expressed as relative response compared to vehicle-treated cells. Values represent mean±SEM derived from at least three independent experiments performed in triplicate. Data were analyzed using Student's t-test. Significance was determined compared to respective untreated control cells, and between ERα and S118A-transfected cells. *, $P < 0.01$; **, $P < 0.001$

## Fig. S6

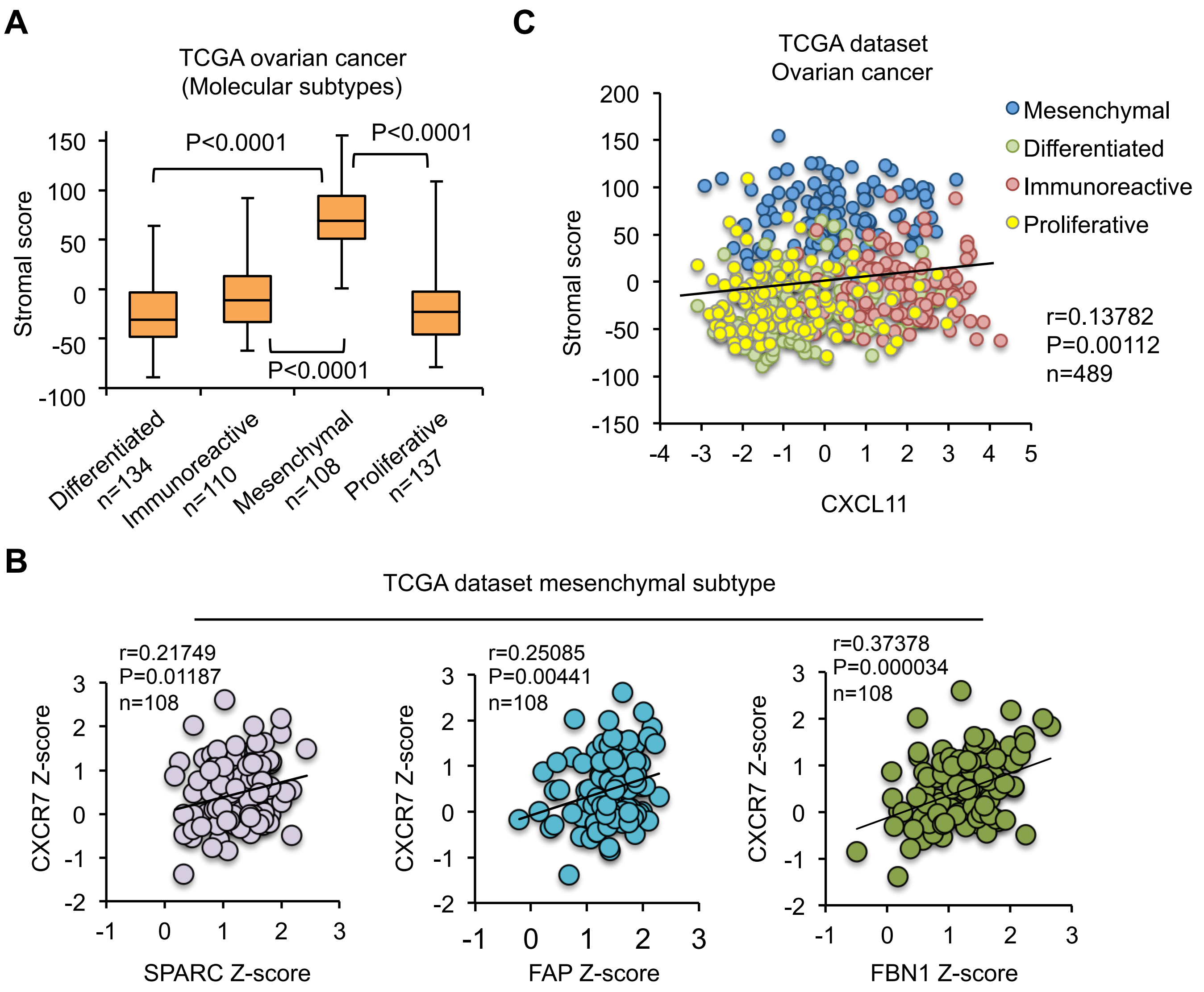


**Fig. S6. CXCR7 and CXCL11 expression is associated with OC stromal signature and markers.**
**A-** Boxplots from GSEA analysis of stromal signature score in the various molecular subtypes of the ovarian TCGA dataset. **B-** Scatter plots of CXCR7 expression relative to stromal markers in the mesenchymal subtypes of the ovarian TCGA dataset. **C-** Pearson correlation analysis of CXCL11 expression with the stromal signature in all four molecular subtypes of the TCGA dataset. Pearson correlation scores (r) and P values are indicated for each.

## Fig. S7

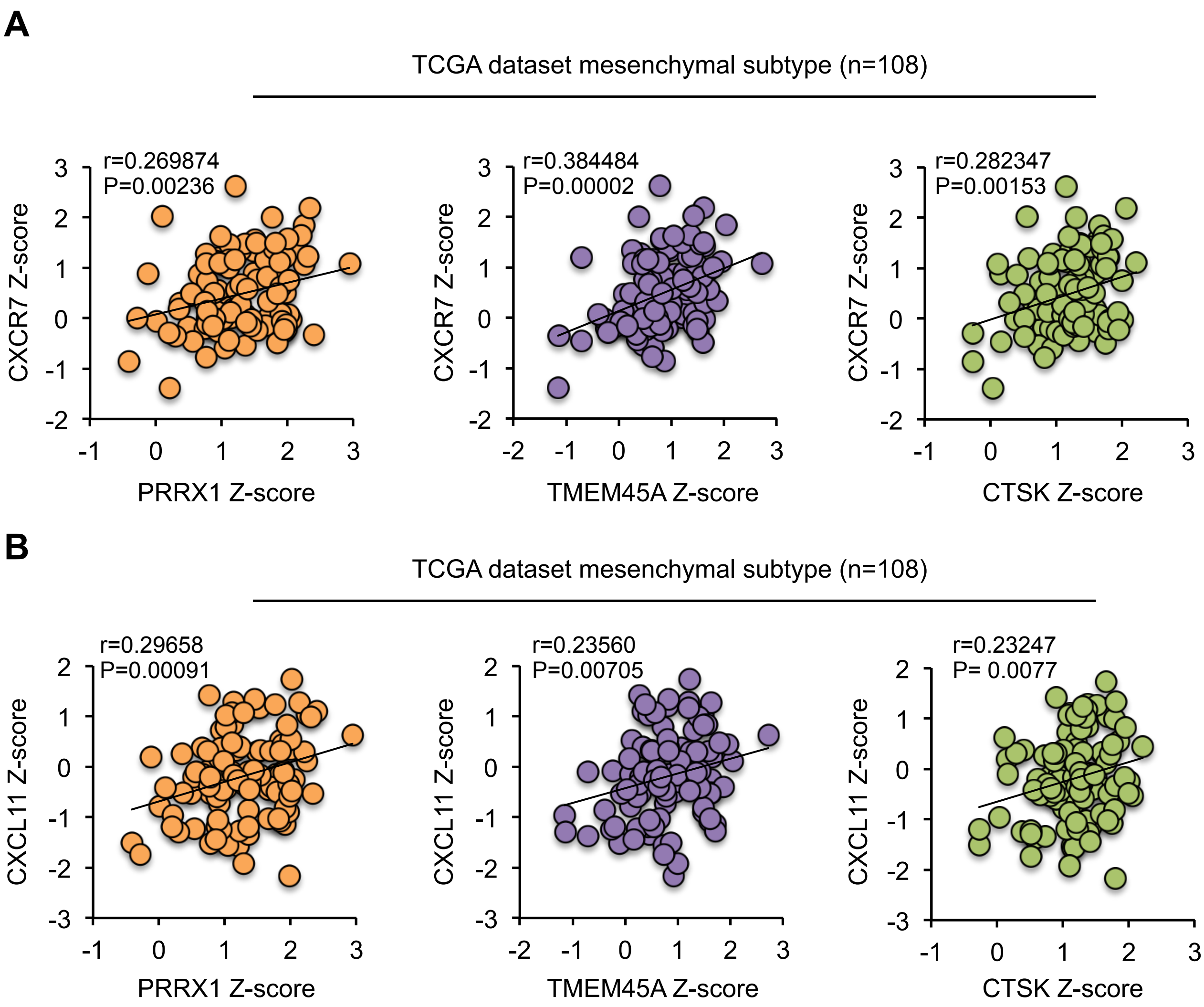


**Fig. S7. CXCR7 and CXCL11 expression correlates with ECM markers.**
**A-** Pearson correlation analysis of CXCR7 expression with markers of extracellular matrix (ECM) remodeling in the mesenchymal subtype of the ovarian TCGA dataset. **B-** Correlation analysis of CXCL11 expression performed as in (A). Pearson correlation scores (r) and P values are indicated for each.

# Fig. S8

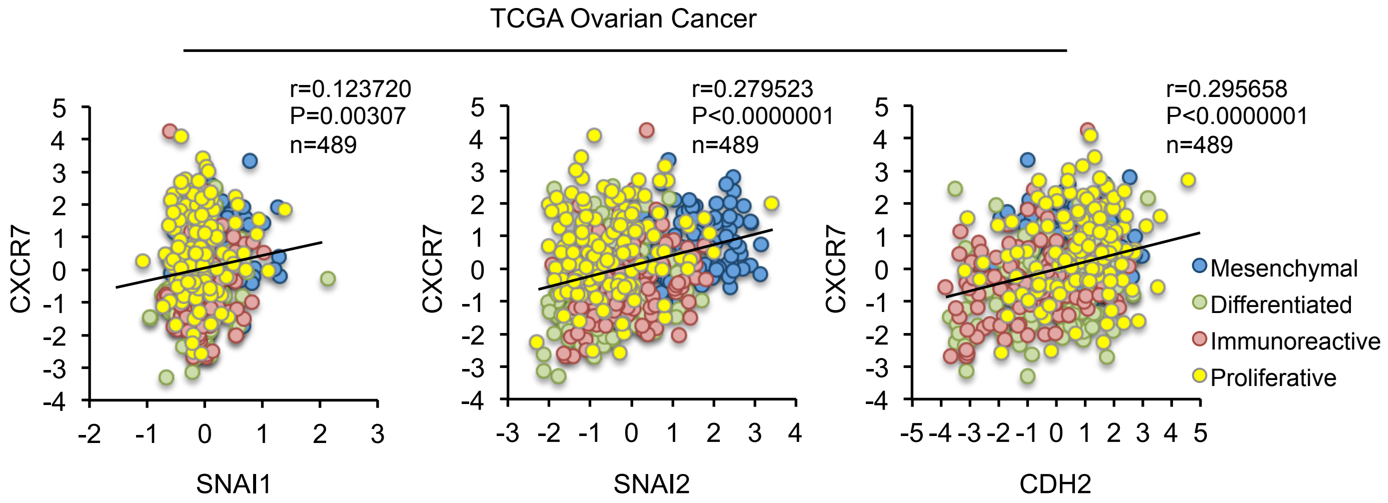


**Fig. S8. CXCR7 expression is associated with EMT indicators.**
**A-** Pearson correlation analysis of CXCR7 expression with markers of epithelial-mesenchymal transition (EMT) in the ovarian TCGA dataset. Each molecular subtypes are color-coded. Pearson correlation scores (r) and P values are indicated for each.
